# Factors affecting safe pesticide-use behaviors among farm plant agriculturists in northeastern Thailand

**DOI:** 10.1186/s12889-024-18662-z

**Published:** 2024-04-20

**Authors:** Bunliang Suphim, Archin Songthap

**Affiliations:** Department of Community Health, Faculty of Public Health, Naresuan University, 99 Village 9, Thapho Subdistrict, Muang District, Phitsanulok Province, 65000 Thailand

**Keywords:** Pesticide use, Safety behaviors, Farm plant agriculturists, Protective motivation theory, Social support

## Abstract

**Background:**

Pesticide poisoning is a major public health problem in Thailand and is the result of intensive inappropriate and unsafe use of pesticides. This analytical cross-sectional study aimed to determine the factors affecting safe pesticide-use behaviors among farm plant agriculturists in northeastern Thailand.

**Methods:**

The study sample included 427 farm plant agriculturists in Loei Province, northeastern Thailand. Individuals were randomly selected by a multistage random sampling technique. The following data were collected via a self-administered questionnaire consisting of 8 parts: (1) sociodemographic characteristics, (2) knowledge about pesticide use, (3) perceived severity of impact from pesticide use among farm plant agriculturists, (4) perceived susceptibility to pesticide use, (5) perceived self-efficacy in the modification of safe pesticide-use behaviors, (6) perceived outcome of the modification of safe pesticide-use behaviors, (7) social support, and (8) safe pesticide-use behaviors. Frequencies, percentages, means, standard deviations, and multiple regression analyses were employed for data analysis.

**Results:**

The majority of participants (79.4%) had high scores for safe pesticide-use behaviors among farm plant agriculturists (scores of 112–150). Factors that significantly affected safe pesticide-use behaviors included knowledge about pesticide use (β = 0.282), social support (β = 0.217), reading information from pesticide labels before pesticide use (β = 0.207), perceived self-efficacy (β = 0.186), female sex (β = -0.140), rice farmer status (β = 0.129), corn farmer status (β = 0.127), perceived susceptibility (β = 0.126), having received information from the internet (β = -0.124), and perceived severity (β = -0.098). Together, these 10 factors were found to explain 32.5% of the safe pesticide-use behaviors among farm plant agriculturists.

**Conclusions:**

Our findings indicate that there is a need to increase the number of promotional activities related to the safe use of pesticides through social support and training, with the aim of increasing the overall level of knowledge, perceived self-efficacy, perceived susceptibility, and modification of the perceived impact severity of pesticide use. Thus, relevant agencies should promote and support the safe use of pesticides by farm plant agriculturists.

**Summary:**

This study revealed that the factors affecting safe pesticide-use behaviors among farm plant agriculturists included knowledge about pesticide use, social support, reading pesticide container labels, perceived self-efficacy in the modification of pesticide-use behaviors, sex, rice farmer status, corn farmer status, perceived susceptibility to pesticide use, having received information from the internet, and perceived severity of impact from pesticide use.

**Supplementary Information:**

The online version contains supplementary material available at 10.1186/s12889-024-18662-z.

## Background

Most of the world’s population is engaged in agriculture. Pesticides are used by farmers to grow more crops by protecting crops from disease, pests, and weeds. As a result, nearly 3 billion kilograms of pesticides are used annually worldwide, including insecticides, herbicides, fungicides, rodenticides, nematicides, molluscicides, plant growth regulators, and other compounds [1, 2]. Thailand is one of five countries in Southeast Asia that uses pesticides [3]. In the 2020–2022 period, Thailand imported approximately 100,000 tons of pesticides per year [4], which suggests that Thai farmers are continuously using pesticides. The top 5 types of imported pesticides used are herbicides, insecticides, fungicides, plant growth regulators, and nematicides [4].

In 1990, the World Health Organization (WHO) estimated that approximately one million unintentional pesticide poisoning events occurred annually, leading to approximately 355,000 deaths globally each year. Such poisoning is strongly related to the unsafe and inappropriate use of pesticides [5]. For examples, farmers are exposed to pesticides either directly or indirectly. By using pesticides on crops, farmers come in direct contact with them, which can affect their organs, such as the skin, eyes, mouth, and respiratory tract, and cause acute reactions, such as dry throat, eye irritation, headache, cough, sneezing, skin rashes, nausea, vomiting, wheezing, and dyspnea. The severity of the effects of these pesticides on one’s health depends upon the exposure time and concentration. In addition, long-term exposure to pesticides increases the concentration of toxins inside organs and can cause chronic diseases such as cancer, neurotoxicity, asthma, necrosis, reproductive disorders, cardiac disease, diabetes, and child neurodevelopmental disorders [2].

The Ministry of Public Health in Thailand (2023) [6] has reported that the incidence of pesticide poisoning is approximately 6,502 cases per year. In northeastern Thailand, the incidence of pesticide poisoning is approximately 4,525 cases per year, which is higher than that in other areas in Thailand. Pesticide poisoning is correlated with disease in Thai farmers. Acute health symptoms, such as coughing (30.6%), red/stinging eyes (26.5%), blurred vision (26.5%), dizziness (25.5%), skin irritation (22.5%), skin rash (21.4%), and chest pain (19.4%), have been reported [7]. Regarding chronic diseases, Juntarawijit and Juntarawijit (2021) [8] reported that the prevalence of diabetes is positively associated with exposure to all types of pesticides, including insecticides, herbicides, fungicides, rodenticides, and molluscicides.

Previous studies have revealed that factors affecting safety behaviors related to pesticide use among farmers include personal characteristics such as sex, age, education, income, farming experience, years of pesticide use, cultivated plants, and training and information about pesticides, such as health care providers, agricultural providers, neighbors, pesticide container labels, the internet, and pesticide retailers [9–15]. Knowledge about pesticide use [13, 16], the perceived severity of the impact of pesticide use among farmers, the perceived susceptibility to pesticide use among farmers, the perceived self-efficacy of prevention from pesticides [11, 17] and social support [18] for modifying pesticide-use behaviors are all important.

Some farmers exhibit unsafe and inappropriate behaviors related to pesticide use, leading to health problems. A previous study indicated that corn farmers in northern Thailand do not always wear masks, gloves, or goggles while using herbicides [19]. Furthermore, some farmers eat food, drink water, drink alcohol, and smoke while using pesticides on their farms [20, 21]. These behaviors can cause health risks resulting from pesticide use among Thai farmers.

Although some previous studies have assessed the factors influencing safe pesticide-use behaviors among farmers, there is little information about these factors in northeastern Thailand. Our study applied the protective motivation and social support theories to determine the factors affecting safe pesticide-use behaviors among farm plant agriculturists. Protective motivation theory helps describe how influencing individuals’ perceptions of health problems can result in changing behaviors to prevent health impacts from pesticide use among farm plant agriculturists. Social support theory helps describe how friends, family, health care providers, or government agencies support knowledge, motivation, personal protective equipment, rewards, etc., in improving safe pesticide-use behaviors. In our study, data were collected from farm plant agriculturists who routinely utilize pesticides. The results of this study can be utilized for creating programs or activities to improve safety behaviors related to pesticide use for farm plant agriculturists to prevent pesticide residue in blood and improve quality of life.

## Methods

### Study design, setting, and participants

This cross-sectional study was conducted to determine the factors affecting safe pesticide-use behaviors among farm plant agriculturists. The population included farm plant agriculturists in Loei Province, northeastern Thailand. The sample included 427 farm plant agriculturists. The sample size was calculated using the estimation of the population mean [22] based on 6,649 people, and the variance (σ^2^) in pesticide-use behavior was found to be 0.36 [23]. We set the error of estimation (e) at 0.035, and the alpha (α) was set at 0.05. The inclusion criteria included (1) age ≥ 20 years, (2) self-mixing or spraying or being hired to spray agrochemicals for at least 6 months, (3) being able to write and read Thai, and (4) being willing to participate in this study. The exclusion criteria included (1) having a sudden illness on the date of data collection and (2) being unable to complete the questionnaire. A multistage random sampling technique was utilized to recruit participants. Four districts out of the 14 in Loei Province were selected as representative districts using a simple random sampling technique. Then, 8 subdistricts out of the 4 districts were selected by simple random sampling. Finally, the participants from each subdistrict were randomly selected using the same technique.

### Research tool

The questionnaire was developed by the researchers based on the theoretical concepts and related studies which composed of 8 parts: (1) sociodemographic characteristics, (2) knowledge about pesticide use, (3) perceived severity of impact from pesticide use, (4) perceived susceptibility to pesticide use, (5) perceived self-efficacy in the modification of pesticide-use behaviors, (6) perceived outcome of the modification of pesticide-use behaviors, (7) social support for modification of pesticide-use behaviors, and (8) safety behaviors related to pesticide use. The sociodemographic characteristics included 15 items regarding data drawn from farm plant agriculturists. Knowledge was assessed through 15 yes/no questions and classified into 3 categories using sum scores [24] of good (score of 10–15), average (score of 5–9), and low (score of 0–4). The perceived severity, perceived susceptibility, and perceived outcome items consisted of 10 5-point Likert scale questions ranging from strongly agree to strongly disagree and were classified into 3 groups [25] using mean scores of 3.68–5.00 for high, 2.34–3.67 for average, and 1.00–2.33 for low. Perceived self-efficacy was assessed with 10 5-point Likert scale questions ranging from strongly believe to strongly disbelieve and was divided into 3 groups [25] according to the mean score: high (3.68–5.00), average (2.34–3.67), and low (1.00–2.33). The social support items consisted of 10 5-point Likert scales ranging from always to never and were divided into 3 groups [25] according to the mean score: high (3.68–5.00), average (2.34–3.67), and low (1.00–2.33). Safe pesticide-use behavior was assessed with 30 5-point Likert scale questions ranging from always to never and was divided into 3 groups [25] according to the mean score: high (3.68–5.00), average (2.34–3.67), and low (1.00–2.33). All questions with an item objective congruence (IOC) index greater than 0.5 were considered to meet the standard criteria for validity. The reliability of the questionnaire was evaluated among 30 respondents who were not included in this study. The reliability test employed Cronbach’s alpha coefficients for all variables except for knowledge, which was assessed by the Kuder-Richardson 20 (KR-20). The reliability scores for knowledge, perceived severity, perceived susceptibility, perceived self-efficacy, perceived outcome, social support, and safe pesticide-use behaviors were 0.71, 0.74, 0.71, 0.75, 0.91, 0.88, and 0.82, respectively.

### Data collection

This study was carried out between September and November 2023. The data were separately collected from the 8 selected subdistricts using a self-administered questionnaire. The data were collected after a period of pesticide spraying. We coordinated with health care providers, community leaders, and village health volunteers to arrange meetings with target farmers. Then, we made an appointment with farmers at the community hall in the villages or meeting rooms of subdistrict administrative organizations or municipalities according to the convenience of each community. We proceeded with clarification about the data collection and distributed questionnaires to the farmers. Participants who met the inclusion criteria and were willing to participate in this study were required to complete the questionnaire, which took approximately 50 min. All questions were checked by researchers for quality. The completed questionnaires were subsequently utilized for data analysis.

### Statistical analysis

The data were analyzed using SPSS version 17, which is a statistical software program. Descriptive statistics, including frequencies, percentages, means and standard deviations, were used to describe sociodemographic characteristics, knowledge, perceived severity, perceived susceptibility, perceived self-efficacy, perceived outcome, social support, and safe pesticide-use behaviors. Stepwise multiple regression analysis was employed to determine the factors that affect pesticide-use behaviors among farm plant agriculturists. All variables chosen for analysis were selected based on the literature review and the theories used in this study. All significance levels were set at 0.05. Before analyzing the data, we examined the variance inflation factor (VIF) value for the multicollinearity test among the independent variables, and none of the variables tested had a value that exceeded 10 [26].

## Results

### Sociodemographic characteristics of the farm plant agriculturists

Among the 427 participants, 50.6% were female, and 34.2% were aged 52–59 years (mean = 50.81, SD = 8.58). The majority of participants (83.4%) were married, and 49.7% had completed primary school. Most of the participants (42.6%) had an average monthly income of more than 10,000 Thai bahts (mean = 8,824.47, SD = 6,671.37), and 62.8% cultivated corn. Most participants (89.0%) used herbicides for pest management, and 59.7% had used pesticides for less than 10 years (mean = 12.32, SD = 8.27). Slightly more than two-thirds (72.4%) mixed and sprayed pesticides themselves. Most of the farm plant agriculturists (85.0%) had received training about safety in pesticide use, and 75.6% had obtained information about pesticides from agricultural providers (Table 1).

**Table 1:** Sociodemographic characteristics of the participants (*n* = 427)

Variables	*n* (%)	Variables	*n* (%)
**Sex**		**Farmers’ use of pesticides**	
Male	211 (49.4)	(can give > 1 answer)	
Female	216 (50.6)	Insecticide	271 (63.5)
**Age (years)**		Herbicide	380 (89.0)
< 40	69 (16.2)	Fungicide	142 (33.3)
40–51	141 (33.0)	Rodenticide	15 (3.5)
52–59	146 (34.2)	**Years of pesticide use**	
≥ 60	71 (16.6)	< 10	255 (59.7)
(Mean = 50.81, SD = 8.58)		10–19	61 (14.3)
**Marital status**		≥ 20	111 (26.0)
Single	30 (7.0)	(Mean = 12.32, SD = 8.27)	
Married	356 (83.4)	**Function in pesticide use**	
Divorced/widowed	41 (9.6)	Only mixer	81 (19.0)
**Education level**		Mixer and sprayer	309 (72.4)
Primary school	212 (49.7)	Only sprayer	37 (8.6)
Junior high school	86 (20.1)	**Training in safe pesticide use**	
High school/equivalent	106 (24.8)	Trained	363 (85.0)
Diploma/equivalent	17 (4.0)	Not trained	64 (15.0)
Bachelor’s degree/higher	6 (1.4)	**Information sources about pesticides** (can give > 1 answer)	
**Average monthly income (Thai baths)**		Health care providers	231 (54.1)
< 5,000	181 (42.4)	Agricultural providers	323 (75.6)
5,000–9,999	64 (15.0)	Relatives	71 (16.6)
≥ 10,000	182 (42.6)	Fellow farmers	138 (32.3)
(Mean = 8,824.47, SD = 6,671.37)		Radio	46 (10.8)
**Cultivated farm plants** (can give > 1 answer)		Community broadcast tower Television	49 (11.5) 88 (20.6)
Sugarcane	64 (15.0)	Internet	71 (16.6)
Corn	268 (62.8)	Pesticide container labels	190 (44.5)
Cassava	128 (30.0)	Pesticide retailers	161 (37.7)
Pineapple	23 (5.4)		
Rice	117 (27.4)		
Tobacco	3 (0.7)		
Peanuts	7 (1.6)		

### Knowledge about pesticide use, perceived severity of impact from pesticide use, perceived susceptibility to pesticide use, perceived self-efficacy in the modification of pesticide-use behaviors, perceived outcome of the modification of pesticide-use behaviors, social support, and safe pesticide-use behaviors

Overall, 95.6% of participants had a high level of knowledge regarding pesticide use, with a mean of 12.28 (SD = 1.69). Similarly, the perceived severity, perceived susceptibility, perceived self-efficacy, perceived outcome, and safe pesticide-use behaviors of the participants were high (63.0%, 73.6%, 94.6%, 89.9%, and 79.4%, respectively), with means of 3.76 (SD = 0.42), 3.87 (SD = 0.41), 4.43 (SD = 0.53), 4.17 (SD = 0.52), and 4.16 (SD = 0.43), respectively. However, the average social support of the participants was 55.0%, with a mean of 3.24 (SD = 0.84) (Table 2).

**Table 2:** Overall knowledge, perceived severity, perceived susceptibility, perceived self-efficacy, perceived outcome, social support, and safe pesticide-use behaviors distributed by level (*n* = 427)

Variables	*n* (%)
**Knowledge regarding pesticide use**	
High (score of 10–15)	408 (95.6)
Average (score of 5–9)	18 (4.2)
Low (score of 0–4)	1 (0.2)
(Mean = 12.28, SD = 1.69)	
**Perceived severity of impact from pesticide use**	
High (3.68–5.00)	269 (63.0)
Average (2.34–3.67)	155 (36.3)
Low (1.00–2.33)	3 (0.7)
(Mean = 3.76, SD = 0.42)	
**Perceived susceptibility to pesticide use**	
High (3.68–5.00)	314 (73.6)
Average (2.34–3.67)	112 (26.2)
Low (1.00–2.33)	1 (0.2)
(Mean = 3.87, SD = 0.41)	
**Perceived self-efficacy in the modification of pesticide-use behaviors**	
High (3.68–5.00)	404 (94.6)
Average (2.34–3.67)	19 (4.4)
Low (1.00–2.33)	4 (1.0)
(Mean = 4.43, SD = 0.53)	
**Perceived outcome of the modification of pesticide-use behaviors**	
High (3.68–5.00)	384 (89.9)
Average (2.34–3.67)	43 (10.1)
(Mean = 4.17, SD = 0.52)	
**Social support**	
High (3.68–5.00)	145 (34.0)
Average (2.34–3.67)	235 (55.0)
Low (1.00–2.33)	47 (11.0)
(Mean = 3.24, SD = 0.84)	
**Safe pesticide-use behaviors**	
High (3.68–5.00)	339 (79.4)
Average (2.34–3.67)	88 (20.6)
(Mean = 4.16, SD = 0.43)	

### Factors affecting safe pesticide-use behaviors among farm plant agriculturists

Stepwise multiple regression analysis was employed to determine the factors that affect safe pesticide-use behaviors among farm plant agriculturists. The VIFs of the independent variables (sex, internet, social support, pesticide container labels, corn, rice, knowledge, perceived self-efficacy, perceived susceptibility, and perceived severity) did not exceed 10 for the multicollinearity test (1.049, 1.111, 1.112, 1.143, 1.200, 1.205, 1.212, 1.328, 1.428, and 1.440, respectively). This result indicated that no associations existed between the independent variables.

The results indicated that participants who had higher mean scores for knowledge, perceived self-efficacy, and perceived susceptibility tended to have better safe pesticide-use behaviors than did those with lower mean scores (β = 0.282, 0.186, and 0.126, respectively). However, participants who had an average mean social support score were more likely to have better safe pesticide-use behaviors than were those with lower mean scores (β = 0.217). Moreover, participants who read information from pesticide labels before pesticide use tended to have better safe pesticide-use behaviors than did those who never read information from pesticide labels (β = 0.207). Similarly, participants who did not grow rice or corn were more likely to have better safe pesticide-use behaviors than were those who grew rice or corn (β = 0.129 and 0.127, respectively). However, female participants were more likely to exhibit unsafe behaviors related to pesticide use than were male participants (β = -0.140), and participants who received information about pesticides from the internet were more likely to exhibit unsafe behaviors related to pesticide use than were those who had not received such information from the internet (β = -0.124). Finally, participants who had higher mean scores for perceived severity were more likely to exhibit unsafe behaviors related to pesticide use than were those who had lower mean scores (β = -0.098). All of these significant factors together explained 32.5% of the safe pesticide-use behaviors among farm plant farmers (adjusted R square = 0.325) (Table 3).

**Table 3:** Factors affecting safe pesticide-use behaviors among farm plant agriculturists (*n* = 427)

Independent variables	b	Beta (β)	*p* value
Knowledge	2.143	0.282	< 0.001
Social support	0.332	0.217	< 0.001
Pesticide container labels (Ref* = no)	5.375	0.207	< 0.001
Perceived self-efficacy	0.454	0.186	< 0.001
Sex (Ref* = male)	-3.598	-0.140	0.001
Rice (Ref* = grow)	3.735	0.129	0.003
Corn (Ref* = grow)	3.386	0.127	0.004
Perceived susceptibility	0.398	0.126	0.009
Internet (Ref* = no)	-4.291	-0.124	0.003
Perceived severity	-0.299	-0.098	0.041

Constant (a) = 59.565, R square = 0.341, adjusted R square = 0.325, F = 21.484, *P* < 0.001, Ref.* = Reference group

## Discussion

Our study aimed to determine the factors that affect safety behaviors related to pesticide use among farm plant agriculturists. The factors that were found to significantly affect safety behaviors related to pesticide use (*p* value < 0.05) were knowledge, social support, reading pesticide container labels, perceived self-efficacy, sex, rice farming, corn farming, perceived susceptibility, obtaining information about pesticides from the internet, and perceived severity.

This study revealed that participants who had higher mean scores of knowledge tended to have better safety behaviors related to pesticide use than did those who had lower mean scores. These findings are consistent with those of previous studies [21, 27, 28]. These studies showed that knowledge regarding pesticides, such as health risks, danger prevention from pesticide use, and appropriate pesticide-use behaviors, is significantly related to safe pesticide-use behaviors among farmers. Hence, if a person expects that knowledge about pesticides can help in safely handling pesticides, then he or she can take immediate action. In other words, knowledge is not only an expectation related to the competency of a person but also a behavior indicator.

Participants who had higher mean social support scores for the modification of pesticide-use behaviors were found to be more likely to have better safe pesticide-use behaviors than were those who had lower mean scores. This finding was similar to that in the study by Lwin et al. (2016) [29], who reported that there is a significant positive correlation between social support and pesticide-use practices. Such social support includes knowledge, motivation, or personal protective equipment received from health care providers/agricultural providers, relatives who are farmers, fellow farmers, or government agencies. Therefore, farmers can change their existing pesticide-use behaviors to safe behaviors. Moreover, social networks have been found to encourage farmers to apply biological pesticides to crops [18].

Our results revealed that participants who read pesticide container labels before use were more likely to have safer pesticide-use behaviors than were those who never read them. These findings mirror those of Tsakiris et al. (2023) [10], who showed that the majority of farmers read information on pesticide labels before using pesticides. This means that obtaining information from pesticide labels significantly increases farmers’ knowledge of pesticide handling and personal protective equipment use and reduces the risk of experiencing health risks related to pesticides [30].

The results also showed that participants who had higher mean scores of perceived self-efficacy tended to exhibit safe pesticide-use behaviors than did those who had lower mean scores. This finding is consistent with that of a study by Oludoye et al. (2023) [11], which indicated that perceived self-efficacy influence farmers’ pesticide safety behaviors. Thus, if farmers expect that safe behaviors involving pesticide use can prevent health risks and health costs, then they take prompt action. Moreover, perceived self-efficacy is also a component of the correlation between knowledge and the modification of behavior related to pesticide use [17].

Male farmers tended to exhibit better safety behaviors related to pesticide use than female farmers did. These findings are in line with those of the study by Tsakiris et al. (2023) [10], which showed that the prevalence of safety behaviors related to pesticide use is lower among females. This means that male farmers use more personal protective equipment, such as face masks, gloves, boots, hats, coveralls, goggles, and respirators, than females do. Therefore, increasing education and implementing training programs for personal protective equipment utilization are important factors in reducing the number of health risks and health costs related to pesticide use among female farmers [31].

The present study showed that participants who did not grow rice or corn tended to exhibit safer pesticide-use behaviors than did farmers who did not grow these crops. These findings are consistent with those of previous studies [32–34]. These studies have revealed that rice and corn farmers exhibit unsafe behaviors related to pesticide use, which can cause pesticides to accumulate in the body. This can cause health impacts and pesticide-related symptoms, including breathlessness, chest pain, dry throat, numbness, muscle weakness, cramps, headache, dizziness, eye irritation, white/red rash, and white/red pimples.

Our results showed that participants who had higher mean scores of perceived susceptibility to pesticide use were more likely to have safe pesticide-use behaviors than were those who had lower mean scores. This finding is consistent with that of a study by Oludoye et al. (2023) [11], which showed that perceived susceptibility influences farmers’ pesticide safety behaviors. Consequently, safe pesticide use can prevent health risks and health costs, and farmers can take action immediately.

Participants who had never obtained information from the internet tended to exhibit safer pesticide-use behaviors than did those who had. These findings are similar to those of Sun et al. (2021) [15], who showed that the use of appropriate pesticides for controlling insects decreases when farmers obtain information from the internet and media. This means that the internet may be a source for disseminating incorrect information regarding pesticide use, which may cause increased health risks during pesticide use. This indicates that the reliability of information sources is important for farmers to consider [29].

Last, participants who had higher mean scores of perceived severity of impact from pesticide use tended to have unsafe behaviors related to pesticide use compared to those who had lower perceived severity. These findings contrast with those of the study by Berni et al. (2021) [35], which showed that perceived severity is positively correlated with safety behaviors in pesticide applicators among farmers. These findings show that there may be other factors, such as awareness, which affect the perceived severity of the impact of pesticide use among farm plant agriculturists. This means that farmers’ awareness of the negative effects of pesticides improves their safety level for pesticide use on crops [36].

In our study, the overall mean scores of knowledge about pesticide use, perceived self-efficacy in the modification of pesticide-use behavior, perceived susceptibility to pesticide use, perceived severity of impact from pesticide use, and safe pesticide-use behaviors among farm plant agriculturists were high. These results indicate that perception is an interrelationship between knowledge and behaviors; i.e., those with high knowledge scores also exhibit good behaviors [28]. In other words, the knowledge of farmers helps to increase their perceptions of and change their behaviors related to safe pesticide use. Moreover, data were collected from farm plant agriculturists who routinely utilize pesticides during their day-to-day lives, which is a strength of our study. Some limitations that might have affected the results are other factors that we did not explore, such as the level of awareness of the negative effects of pesticides on health, and the evaluation of the use of policies, measures, and projects that aim to reduce the use of hazardous chemicals and promote the correct use of chemicals, such as safe use, Integrated Pest Management (IPM), and Good Agricultural Practice (GAP). These unexplored factors might be associated with safe pesticide-use behaviors among farm plant agriculturists in the Thai social context.

## Conclusion and suggestions

Our study revealed that knowledge about pesticide use, perceived self-efficacy in the modification of pesticide-use behavior, perceived susceptibility to pesticide use, and perceived severity of impact from pesticide use among farm plant agriculturists is high. These factors help to increase farmers’ behaviors related to safe pesticide use to a high degree. Moreover, other factors affect safe pesticide-use behaviors, including social support, reading pesticide container labels, sex, rice farmer status, corn farmer status, and receiving information from the internet. As a result, health care providers/agricultural providers should increase the number of promotional activities related to the safe use of pesticides through social support and training, with the aim of increasing the overall level of knowledge regarding reading pesticide labels before pesticide use, perceived self-efficacy, perceived susceptibility, and modification of the perceived severity of the impact of pesticide use, especially for agriculturists who are rice and corn farmers. Furthermore, relevant agencies should promote and support the safe use of pesticides by farm plant agriculturists. Further studies should examine awareness, with the aim of determining whether this factor influences safe pesticide-use behaviors among farm plant agriculturists. In addition, evaluations of the use of policies, measures, and projects to reduce the use of hazardous chemicals and promote the correct use of chemicals, such as safe use, Integrated Pest Management (IPM), and Good Agricultural Practice (GAP), are needed in Loei Province, northeastern Thailand.

### Electronic supplementary material

Below is the link to the electronic supplementary material.

Supplementary Material 1

## Data Availability

The data that support the findings of this study are available from Naresuan University Research Ethics Committee but restrictions apply to the availability of these data, which were used under license for the current study, and so are not publicly available. Data are however available from the authors upon reasonable request and with permission of Naresuan University Research Ethics Committee.
