# Supplementary material for: Factors affecting safe pesticide-use behaviors among farm plant agriculturists in northeastern Thailand
Source: BMC Public Health. 2024 Apr 20;24:1096. doi: 10.1186/s12889-024-18662-z (PMC11032588; doi:10.1186/s12889-024-18662-z)
Supplement: Supplementary file 1 — Supplementary Material 1 [file 12889_2024_18662_MOESM1_ESM.docx]

**Research questionnaire**

**Factors affecting safe pesticide-use behaviors among farm plant agriculturists in northeastern Thailand**

This questionnaire composes of 8 parts:

Part 1: Sociodemographic characteristics

Part 2: Knowledge about pesticide use

Part 3: Perceived severity of impact from pesticide use

Part 4: Perceived susceptibility to pesticide use

Part 5: Perceived self-efficacy in the modification of pesticide-use behaviors

Part 6: Perceived outcome of the modification of pesticide-use behaviors

Part 7: Social support for the modification of pesticide-use behaviors

Part 8: Safe pesticide-use behaviors

**Part 1: Sociodemographic characteristics**

**Instructions: Please mark with a ✓ in ❑ or fill in the blank with information that reflects your situation accurately.**

1. Gender

❑ 1. Male ❑ 2. Female

2. Age…………….years

3. Marital Status

❑ 1. Single ❑ 2. Married ❑ 3. Divorced

❑ 4. Widowed

4. Education Level

❑ 1. Primary school ❑ 2. Junior high school ❑ 3. High school/equivalent

❑ 4. Diploma/equivalent ❑5. Bachelor’s degree/higher

❑ 6. Other (Please specify): ………………………………..……..

5. Average monthly income: ……………………baht

6. Cultivated farm plants (can give >1 answer)

❑ 1. Sugarcane ❑ 2. Corn ❑ 3. Cassava

❑ 4. Pineapple ❑5. Other (Please specify): ……………………………

7. Ownership of the land to cultivate farm plants

❑ 1. Own land ❑ 2. Rent land

8. Amount of cultivated farm plant area: ………………Rai (1,600 square meters)

9. Time period for cultivating farm plants: ………………years

10. Types of pesticides used (can give >1 answer)

❑ 1. Insecticide ❑ 2. Herbicide ❑ 3. Fungicide

❑ 4. Rodenticide ❑5. Other (Please specify): ……………………………

11. Length of time of pesticide use: ……years (>6 months counted as 1 year)

12. Your duty in using pesticides

❑ 1. Only the mixer ❑ 2. Mixer and sprayer ❑ 3. Only the sprayer

13. Cost of pesticide use in one time: ……………….baht

14. Have you ever received any training on pesticides?

❑ 1. Yes ❑ 2. No

15. Where have you received information about pesticides? (can give >1 answer)

❑ 1. Health care provider ❑ 2. Agriculturalist ❑ 3. Relatives

❑ 4. Fellow farmers ❑ 5. Radio ❑ 6. Community broadcast tower

❑ 7. Television ❑ 8. Internet ❑ 9. Pesticide container labels

❑ 10. The pesticide retailers ❑11. Other (Please specify): ……………………………

**Part 2: Knowledge about pesticide use**

**Instructions: Please mark (✓) if the statement is correct and with mark (🞫) if the statement is incorrect.**

.............1. When purchasing pesticides, you should choose the type than has high toxicity and remaining for a long time will prevent the need to spray pesticides frequently.

.............2. When purchasing pesticides, you should choose them according to the advice of healthcare providers.

.............3. When mixing pesticides. you must use a stick or other device for stirring instead of using your hands.

.............4. All types of pesticides can be mixed together.

.............5. When mixing pesticides, you must wear personal protective equipment (PPE), i.e., gloves and a face mask.

.............6. You must check the readiness of tools and equipment before spraying pesticides.

.............7. Pesticides should be sprayed under strong winds and high temperatures.

.............8. When spraying pesticides, you should not eat food, drink water, smoke cigarette, or drink alcohol.

.............9. If the nozzle is clogged while spraying pesticides, you can use mouthpiece.

............10. When spraying pesticides, they must be upwind.

........... 11. Pesticide containers or pesticide spraying equipment can be cleaned in natural water sources.

............12. After pesticide spraying, you must change your clothes, and take a shower.

............13. The disposal of empty pesticide containers should be properly managed by the relevant agency.

............14. After spraying pesticides, warning signs should be posted prohibiting entering areas where pesticides are sprayed.

............15. You should store pesticides in separate places away from children, older adults, and animals.

**Part 3: Perceived severity of impact from pesticide use**

**Instructions: How do you feel about the following statements?**

| Statement | Strongly agree | Agree | Undecided | Disagree | Strongly disagree |
| --- | --- | --- | --- | --- | --- |
| 1. Spraying pesticides without wearing a mask can cause eye irritation and stinging. |  |  |  |  |  |
| 2. Using bare hands to mix pesticides can be done without irritation, dermatitis, and itchy skin. |  |  |  |  |  |
| 3. Exposure to pesticides in the body can cause chest pain. |  |  |  |  |  |
| 4. Using a mouthpiece sprayer nozzle can be done without causing nausea and vomiting. |  |  |  |  |  |
| 5. Long-term exposure to pesticides can cause impaired memory. |  |  |  |  |  |
| 6. Pesticides are toxic to the pancreas and may induce diabetes. |  |  |  |  |  |
| 7. Exposure to pesticides could increase chances of having a miscarriage, a baby with defects, or other problems. |  |  |  |  |  |
| 8. Long-term exposure to pesticides can cause cancer. |  |  |  |  |  |
| 9. Keeping pesticide containers at home can cause weakness and easily to get sick due to exposure to long-term pesticides. |  |  |  |  |  |
| 10. Using pesticides can cause chronic illness, loss of time, and a lot of costs for medical care. |  |  |  |  |  |

**Part 4: Perceived susceptibility to pesticide use**

**Instructions: How do you feel about the following statements?**

| Statement | Strongly agree | Agree | Undecided | Disagree | Strongly disagree |
| --- | --- | --- | --- | --- | --- |
| 1. Mixing pesticides with an empty hand increase the risk of causing inflammation, rashes, itching, burning, discoloration of the nails, and numbness of the tips of the hands. |  |  |  |  |  |
| 2. Using your mouth to open a pesticide container does not cause nausea, vomiting, fatigue, chest tightness, and suffocation. |  |  |  |  |  |
| 3. Mixing pesticides to the dose indicated on the pesticide label reduces the risk of danger to the brain, eyes, skin, and respiratory system. |  |  |  |  |  |
| 4. Using many types of pesticides increase the risk of danger to the brain, eyes, skin, and respiratory system. |  |  |  |  |  |
| 5. Not wearing a face mask during pesticide increase the risk of causing eye irritation, eye pain, chest tightness, dry throat, and coughing. |  |  |  |  |  |
| 6. Eating, drinking, smoking, or alcohol drinking during pesticide spraying increase a risk of causing diabetes, and cardiovascular disease. |  |  |  |  |  |
| 7. Wearing normal clothes during pesticide spraying reduces the risk of burning sensations and skin irritation. |  |  |  |  |  |
| 8. Posting warning signs in pesticide spraying areas can help prevent people from entering pesticide spraying areas and reduce the risk of harm to the brain, eyes, skin, and respiratory system. |  |  |  |  |  |
| 9. Changing clothes and taking a shower after pesticide spraying can reduce irritation, burning, itchy, and rashes on the skin. |  |  |  |  |  |
| 10. Used pesticide containers can be cleaned and reused pesticides penetrating to the body. |  |  |  |  |  |

**Part 5: Perceived self-efficacy in the modification of pesticide-use behaviors**

**Instructions: How much do you believe your ability to perform the following practices?**

| Statement | Strongly believe | Believe | Undecided | Disbelieve | Strongly disbelieve |
| --- | --- | --- | --- | --- | --- |
| 1. Read the instructions written on the container before applying the pesticide. |  |  |  |  |  |
| 2. Wear gloves or a face mask while mixing the pesticides. |  |  |  |  |  |
| 3. Check the readiness of pesticide spraying equipment before use. |  |  |  |  |  |
| 4. Wear a long-sleeved shirt and long-legged pants while spraying pesticides. |  |  |  |  |  |
| 5. Do not smoke, drink, or eat while spraying. |  |  |  |  |  |
| 6. Do not mouthpiece on the equipment sprayer when it is clogged. |  |  |  |  |  |
| 7. Do not allow the children or older adults or other persons or pets to enter the farm while spraying. |  |  |  |  |  |
| 8. After spraying, change your clothes and take a shower. |  |  |  |  |  |
| 9. Do not take used pesticide containers back and throw them away with another household waste. |  |  |  |  |  |
| 10. Do not collect crops for sale or eat them before the period recommended on the pesticide label. |  |  |  |  |  |

**Part 6: Perceived outcome of the modification of pesticide-use behaviors**

**Instructions: How do you feel in managing the following practices?**

| Statement | Strongly agree | Agree | Undecided | Disagree | Strongly disagree |
| --- | --- | --- | --- | --- | --- |
| 1. If you read the label before mixing pesticides, it will help you not be harmed by pesticides. |  |  |  |  |  |
| 2. If you wear gloves and an eye mask while mixing pesticides, it will help you not be harmed by pesticides. |  |  |  |  |  |
| 3. If you have checked the readiness of the equipment before spraying chemicals, it will help you not be harmed by pesticides. |  |  |  |  |  |
| 4. If you wear a long-sleeved shirt and long-legged pants resistance chemicals, it will help you not be harmed by pesticides. |  |  |  |  |  |
| 5. If you do not smoke, alcohol drink, drink, or eat while spraying, it will help you not be harmed by pesticides. |  |  |  |  |  |
| 6. If you do not mouthpiece on the equipment sprayer when it is clogged, it will help you not be harmed by pesticides. |  |  |  |  |  |
| 7. If you do not spray pesticides while there is strong wind and the sun is not strong, it will help keep you safe from pesticides. |  |  |  |  |  |
| 8. If you change clothes and take a shower after pesticide spraying, it will help keep you safe from pesticides. |  |  |  |  |  |
| 9. If you post warning signs in pesticide spraying areas, it can help prevent pesticide exposure to children, older adults, and other people entering pesticide spraying areas. |  |  |  |  |  |
| 10. If you do not collect crops for sale or eat them before the period recommended on the pesticide label, it can help not be dangerous from pesticides. |  |  |  |  |  |

**Part 7: Social support for the modification of pesticide-use behaviors**

**Instructions: How often do you receive support on the modification of pesticide-use behaviors?**

| Statement | Always | Often | Sometimes | Rarely | Never |
| --- | --- | --- | --- | --- | --- |
| Emotional support |  |  |  |  |  |
| 1. You receive follow-up, attentive, and home visits from healthcare providers and agricultural officers to provide information about safe pesticides and safe practices. |  |  |  |  |  |
| 2. You receive praise, respect, and encouragement from relatives and fellow farmers when you perform safe practices in pesticide use. |  |  |  |  |  |
| 3. You receive praise, respect, and encouragement from healthcare providers and agricultural officers when you perform safe practices in pesticide use. |  |  |  |  |  |
| Material and labor support |  |  |  |  |  |
| 4. You receive personal protective equipment (PPE) for preparation and spraying pesticides from the government. |  |  |  |  |  |
| 5. You receive from relatives and fellow farmers in safety pesticide use. |  |  |  |  |  |
| Information Support |  |  |  |  |  |
| 6. You receive information about safe pesticide use from healthcare providers and agricultural officers. |  |  |  |  |  |
| 7. You receive information about safe pesticide use from relatives and fellow farmers. |  |  |  |  |  |
| 8. You receive information about the reduction in pesticide use from healthcare providers and agricultural officers. |  |  |  |  |  |
| Appraisal Support |  |  |  |  |  |
| 9. You receive support and recommendations about safe pesticide use from healthcare providers and agricultural officers. |  |  |  |  |  |
| 10. You receive a warning from relatives or fellow farmers about safe pesticide use. |  |  |  |  |  |

**Part 8: Safe pesticide-use behaviors**

**Instructions: How often do you practice safe pesticide-use behaviors?**

| Statement | Always | Often | Sometimes | Rarely | Never |
| --- | --- | --- | --- | --- | --- |
| Safety behavior while preparing pesticide use |  |  |  |  |  |
| 1. You chose to use pesticides that have less toxicity. |  |  |  |  |  |
| 2. You chose to purchase pesticides recommended by relatives or fellow farmers. |  |  |  |  |  |
| 3. You read the instructions written on the container before using the pesticides. |  |  |  |  |  |
| 4. You mix pesticides higher dose than indicated on the pesticide label. |  |  |  |  |  |
| 5. You wear gloves while mixing pesticides. |  |  |  |  |  |
| 6. You use your mouth to open the pesticide containers. |  |  |  |  |  |
| 7. You wear a face mask while mixing pesticides. |  |  |  |  |  |
| 8. You use a stir stick or equipment when mixing pesticides. |  |  |  |  |  |
| 9. You mix pesticides in a place near children, older adults, or other people. |  |  |  |  |  |
| 10. You check the pesticide equipment if they are not leaking before spraying. |  |  |  |  |  |
| Safety behavior while pesticide use. |  |  |  |  |  |
| 1. You wear a face mask while spraying. |  |  |  |  |  |
| 2. You wear a long-sleeved shirt and long-legged pants while spraying pesticides. |  |  |  |  |  |
| 3. You smoke or drink alcohol while spraying. |  |  |  |  |  |
| 4. You stand upwind while spraying pesticides. |  |  |  |  |  |
| 5. You spray at the right time of day (evening and in the morning) and avoid sunny periods. |  |  |  |  |  |
| 6. You do not eat while spraying. |  |  |  |  |  |

**Part 8: Safe pesticide-use behaviors (Cont.)**

**Instructions: How often do you practice safe pesticide-use behaviors?**

| Statement | Always | Often | Sometimes | Rarely | Never |
| --- | --- | --- | --- | --- | --- |
| 7. You spray pesticides when children, older adults, other people, or animals are near the farm. |  |  |  |  |  |
| 8. You do not use equipment leaking in spraying. |  |  |  |  |  |
| 9. You use a mouthpiece on the equipment sprayer when it is clogged. |  |  |  |  |  |
| 10. You do not drink while spraying. |  |  |  |  |  |
| Safety behavior after pesticide use. |  |  |  |  |  |
| 1. You do not allow children, older adults, other persons, or animals to enter the farm while spraying. |  |  |  |  |  |
| 2. You change clothes and take a shower after spraying pesticides, |  |  |  |  |  |
| 3. You wash clothes used for spraying separately from ordinary clothes. |  |  |  |  |  |
| 4. You take the empty pesticide containers back to the relevant agency to be properly managed. |  |  |  |  |  |
| 5. You wash pesticide containers or pesticide spraying equipment separately from ordinary containers. |  |  |  |  |  |
| 6. You collect crops for sale or eat them before the period recommended on the pesticide label. |  |  |  |  |  |
| 7. You dispose the empty of pesticide containers to prevent reuse. |  |  |  |  |  |
| 8. Do not pour water used for cleaning pesticide spraying equipment into water sources or residential areas. |  |  |  |  |  |
| 9. You bring used containers of pesticides back and throw them away with another household waste. |  |  |  |  |  |
| 10. You have warning signs posted in areas where pesticides are sprayed. |  |  |  |  |  |
